# LncRNA CDKN2BAS predicts poor prognosis in patients with hepatocellular carcinoma and promotes metastasis via the miR-153-5p/ARHGAP18 signaling axis

**DOI:** 10.18632/aging.101645

**Published:** 2018-11-29

**Authors:** Junzheng Chen, Xitian Huang, Weijun Wang, Hongcheng Xie, Jianfeng Li, Zhenfen Hu, Zhijian Zheng, Huiyong Li, Lingfang Teng

**Affiliations:** 1 Surgical Center, The Affiliated Wenling Hospital of Wenzhou Medical University, Wenling 317500, Zhejiang Province, China; 2 Department of Hepatology, The Affiliated Wenling Hospital of Wenzhou Medical University, Wenling 317500, Zhejiang Province, China; 3 Department of Hepatobiliary Surgery, Sanxinmeide Geriatrics Hospital of Wenling, Wenling 317500, Zhejiang Province, China; * Equal contribution

**Keywords:** hepatocellular carcinoma (HCC), long noncoding RNA (lncRNA), CDKN2BAS, ARHGAP18, miR-153-5p

## Abstract

Background: Growing evidence shows that long noncoding RNAs (lncRNAs) play a crucial role in cancer progression. However, whether lncRNA CDKN2BAS is involved in human hepatocellular carcinoma (HCC) metastasis remains unclear.

Methods: Human lncRNA microarray analysis was performed to detect differential expression levels of lncRNAs in metastatic HCC tissues. Effects of CDKN2BAS on cell proliferation, migration, and apoptosis were determined by MTT assay, colony formation assay, migration assay, scratch assay, and flow cytometry. The xenograft experiment was used to confirm the effect of CDKN2BAS on HCC *in vivo*. qRT-PCR and Western blot were performed to determine the expression levels of mRNAs and proteins. Luciferase reporter assay was used to identify the specific target relationships.

Results: CDKN2BAS was remarkably up-regulated in metastatic HCC tissues compared with the adjacent non-tumor tissues. CDKN2BAS promotes HCC cell growth and migration *in vitro* and *in vivo*. Additionally, CDKN2BAS upregulated the expression of Rho GTPase activating protein 18 (ARHGAP18) by sponging microRNA-153-5p (miR-153-5p), and thus promoted HCC cell migration. Besides, CDKN2BAS downregulated the expression of Krüppel-like factor 13 (KLF13) and activated MEK-ERK1/2 signaling, thus reducing apoptosis in HCC cells.

Conclusions: Our study revealed that lncRNA CDKN2BAS promotes HCC metastasis by regulating the miR-153-5p/ARHGAP18 signaling.

## Introduction

Hepatocellular carcinoma (HCC) is the sixth most common cancer worldwide and also one of the leading causes of cancer-related death [1,2]. Significant advances have been achieved in the diagnosis and treatment of HCC, but the long-term survival rate of patients with HCC is still low [3]. Poor prognosis of HCC is mainly due to two reasons, the cancer recurrence and the metastasis/development of primary tumors in the remaining liver [4]. Potentially biological mechanisms in patients with HCC could be different. Little is known about the risk factors for intrahepatic metastasis and early recurrence after hepatectomy [5-7]. Cancer metastasis is a complicated process involves a variety of genetic changes [8]. Signaling pathways such as transforming growth factor-β (TGF-β), vascular endothelial growth factor (VEGF), Wnt/β-catenin, MAPK, and small G protein pathway, play important roles in the development of HCC [9-11]. Currently, increasing attention has been paid to the collaboration of diverse factors in the occurrence and progression of HCC, including miRNA, long non-coding RNA (lncRNA), and epigenetic factors [12].

LncRNAs are a class of endogenous RNA molecules longer than 200 nucleotides in length [13-15]. In the past few years, the importance of lncRNAs in the metastasis of HCC has been documented [16-19]. CDKN2BAS, a member of the lncRNA family, plays important roles in cell proliferation, apoptosis, and extra-cellular matrix remodeling [20,21]. CDKN2BAS expression has been reported to be associated with susceptibility to several important human diseases, such as coronary artery disease and diabetes [20]. CDKN2BAS has also been declared to be involved in brain tumor, breast cancer, and medulloblastoma [21–23]; however, the biological function of CDKN2BAS in HCC remains unclear. In this study, we investigated the role of CDKN2BAS in HCC metastasis and also explored the underlying mechanism.

## RESULTS

### CDKN2BAS expression is up-regulated in the metastatic HCC tissues and cell lines

**Figure 1:**
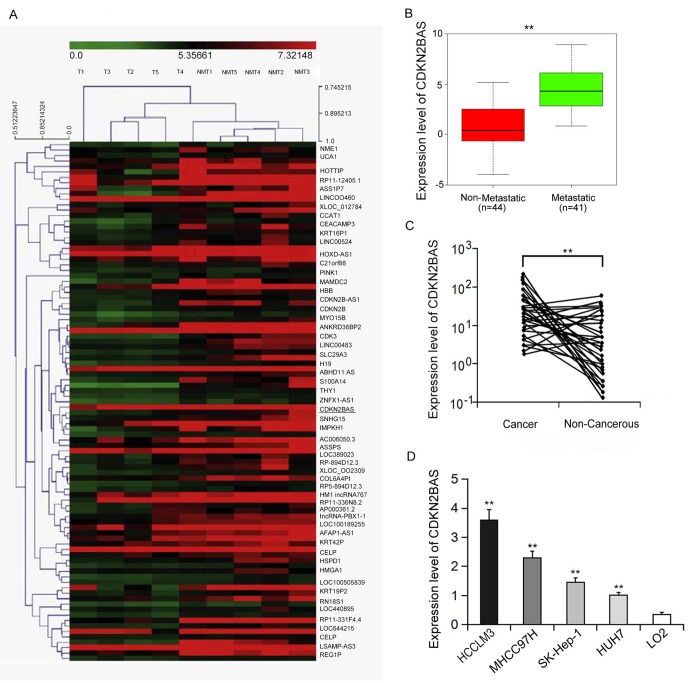
**CDKN2BAS is associated with HCC metastasis.** (**A**) Expression profiles of LncRNA and mRNA from two groups of HCC tissue samples; (**B**) The expression of CDKN2BAS in metastatic and non-metastatic HCC tissues; (**C**) The expression of CDKN2BAS in cancer and paired adjacent non-cancer tissues of the metastatic group; (**D**) The expression of CDKN2BAS in human HCC cell lines. **P < 0.01.

Human gene expression array was utilized to analyze the differential expression levels of lncRNAs in the metastatic and non-metastatic HCC tissues. As shown in Figure 1A, the hierarchical clustering analysis found that 137 non-coding RNA transcripts were expressed differently between two groups (over 2-fold, *P* < 0.01). CDKN2BAS is the most differentially expressed lncRNA, whose expression was 10 times higher than that in the non-metastatic group, implying that CDKN2BAS may be involved in the progression and metastasis of HCC. We further verified the expression of CDKN2BAS in HCC tissues (n=85) by qRT-PCR. The results showed that the expression of CDKN2BAS in the metastatic group was significantly higher than that in the non-metastatic group (Figure 1B). In addition, CDKN2BAS expression was up-regulated in most of the cancer tissues compared with the non-cancer tissues in the metastatic group (Figure 1C). Moreover, CDKN2BAS expression was highly related to the metastatic ability of HCC cell lines (Figure 1D). Together, these results indicate that CDKN2BAS is involved in HCC metastasis.

### CDKN2BAS promotes HCC cell migration both *in vivo* and *in vitro*

**Figure 2:**
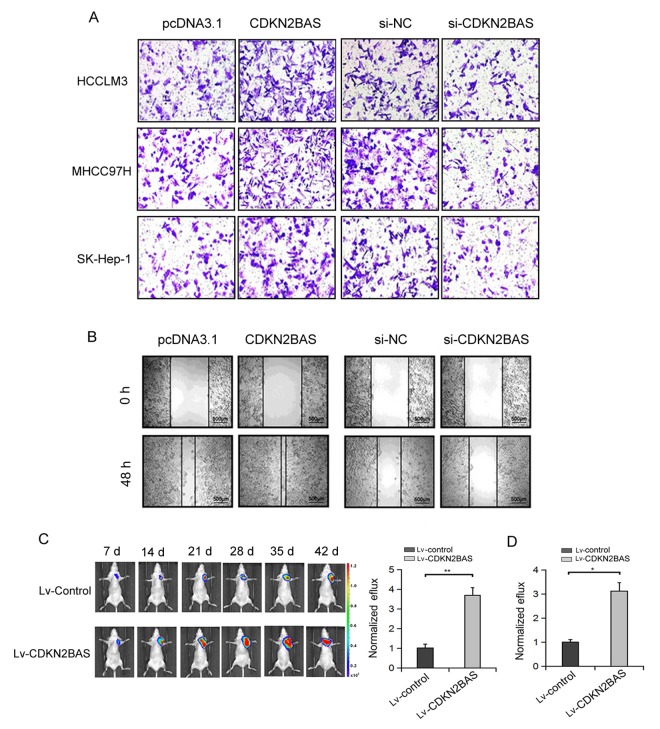
**The effect of CDKN2BAS on HCC cell migration *in vitro* and *in vivo*.** (**A**) The effect of CDKN2BAS on HCC cell migration was detected by Transwell migration assay; (**B**) The effect of overexpression or silencing of CDKN2BAS on HCCLM3 cell migration was detected by Scratch assay; (**C**) CDKN2BAS promoted the metastasis of HCCLM3 cells in nude mice. Left panel: representative images of lung metastasis were captured by bioluminescence imaging. Right panel: Luciferase signal intensities of nude mice 6 week after injection with HCCLM6/CDKN2BAS cells or control cells. (**D**) CDKN2BAS promoted the metastasis of MHCC97H cells in nude mice. Luciferase signal intensities of nude mice 6 week after injection with MHCC97H/CDKN2BAS cells or control cells. *P < 0.05, **P < 0.01.

Next, we determined the effect of CDKN2BAS on HCC cell migration. Transwell migration assay showed that the migration ability of HCC cells was increased over 2-fold after CDKN2BAS overexpression, whereas the migration ability of HCC cells was significantly inhibited after CDKN2BAS knockdown (Figure 2A). Scratch assay confirmed the pro-metastatic role of CDKN2BAS in MHCC97H cells (Figure 2B). To examine the effect of CDKN2BAS *in vivo*, we used a bioluminescent mouse model. The results showed that overexpression of CDKN2BAS significantly increased the bioluminescent signals in mice injected with HCCLM3 or MHCC97H cells (Figure 2C-D). These results indicate that CDKN2BAS exerts a promotional role in HCC metastasis.

### CDKN2BAS promotes HCC cell growth via inhibiting apoptosis

**Figure 3:**
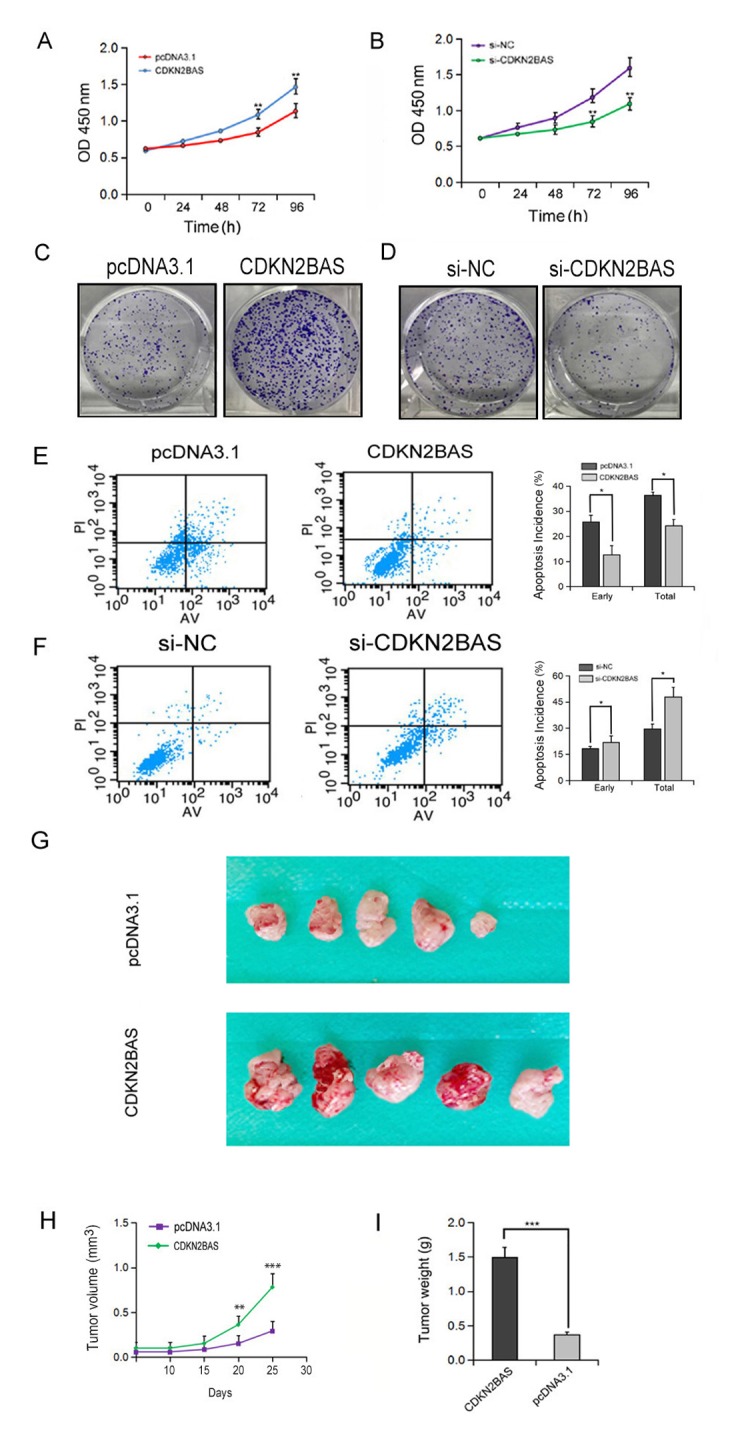
**The effect of CDKN2BAS on HCC cell growth *in vitro* and *in vivo*.** (**A**-**B**) The effect of overexpression or knockdown of CDKN2BAS on the growth of HCCLM3 cells was detected by MTT assay; (**C**-**D**) The effect of overexpression or knockdown of CDKN2BAS on the colony formation of HCCLM3 cells was detected by colony formation assay; (**E**-**F**) The effect of CDKN2BAS on EPI-induced apoptosis in HCCLM3 cells was detected by flow cytometry; (**G**) Tumor xenograft model in nude mice. CDKN2BAS-transfected HCCLM3 cells and control cells were inoculated to the right side of nude mice. (**H**-**I**) The tumor volume and tumor weight were analyzed. *P < 0.05, **P < 0.01, ***P < 0.001.

To detect the effect of CDKN2BAS on HCC cell growth, MTT assay was carried out. The results showed that overexpression of CDKN2BAS significantly increased the growth of MHCC97H cells, whereas knockdown of CDKN2BAS significantly inhibited cell growth (Figure. 3A-B). In agreement with these results, colony formation assay showed that CDKN2BAS overexpression significantly increased the colony number of MHCC97H cells, while opposite result was observed upon CDKN2BAS knockdown (Figure. 3C-D). Moreover, ectopic overexpression of CDKN2BAS significantly inhibited the apoptotic rate of HCC cells after EPT treatment (Figure 3E). In contrast, CDKN2BAS knockdown enhanced EPI-induced apoptosis (Figure 3F). In addition, the tumor xenograft model of MHCC97H cells in nude mice showed that overexpression of CDKN2BAS significantly increased the tumor volume and tumor weight (Figure 3G-I). Collectively, these results suggest that CDKN2BAS plays an oncogenic role in HCC.

### CDKN2BAS up-regulates ARHGAP18 expression via competitively binding with miR-153-5p

**Figure 4:**
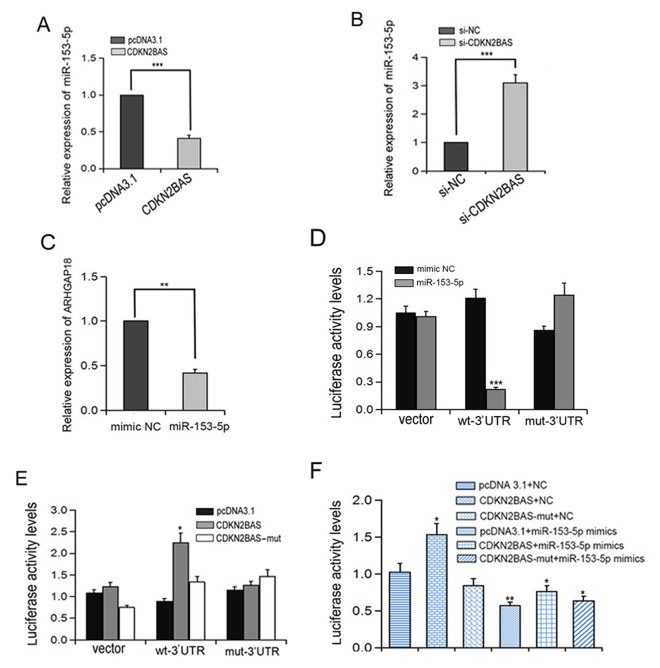
**Interaction between CDKN2BAS and miR-153-5p regulates the expression of ARHGAP18.** (**A**-**B**) The relative expression of miR-153-5p was detected by qRT-PCR after CDKN2BAS overexpression or knockdown; (**C**) The expression of ARHGAP18 was detected by qRT-PCR after miR-153-5p treatment; (**D**-**F**) Luciferase activity was detected in HCCLM3 cells. *P < 0.05, **P < 0.01, ***P < 0.001.

LncRNA-miRNA and mRNA-miRNA interactions are generally associated with a variety of biological processes. We found that CDKN2BAS has a predicted targeting binding site of miR-153-5p (http://www.targetscan.org/vert_72/). We speculated that CDKN2BAS played a competitive role as endogenous RNA (ceRNA) by sponging miR-153-5p during HCC metastasis. To test this hypothesis, we first determined the effect of CDKN2BAS on miR-153-5p expression. We found that overexpression of CDKN2BAS decreased miR-153-5p expression, whereas knockdown of CDKN2BAS increased miR-153-5p expression (Figure 4A-B). Interestingly, the expression of ARHGAP18 was down-regulated after miR-153-5p mimics transfection (Figure 4C). To determine the relationship between miR-153-5p and ARHGAP18, ARHGAP18 3’-UTR regions containing the WT binding site of miR-153-5p (ARHGAP18 wt-3’UTR) or the mutant binding site of miR-153-5p (ARHGAP18 mut-3’UTR) were inserted into the luciferase reporter vector. Luciferase reporter assay showed that miR-153-5p inhibited the luciferase activity of ARHGAP18 wt-3’UTR but had no effect on the ARHGAP18 mut-3’UTR (Figure 4D). In addition, CDKN2BAS overexpression significantly increased the luciferase activity of ARHGAP18 wt-3’UTR, but overexpression of CDKN2BAS-mut did not have this effect (Figure 4E). Moreover, transfection with miR-153-5p eliminated the increased luciferase activity of ARHGAP18 wt-3’UTR induced by CDKN2BAS (Figure 4F). Together, these data suggest CDKN2BAS acts as a ceRNA of miR-153-5p.

### Down-regulation of ARHGAP18 inhibits HCC cell migration

**Figure 5:**
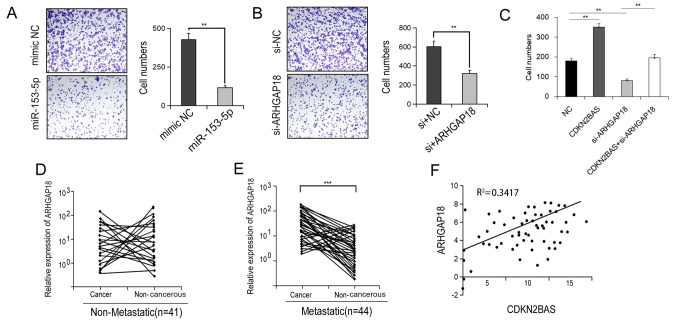
**ARHGAP18 promotes the metastatic phenotype of HCC.** (**A**-**B**) Transwell migration assay was performed in the miR-153-5p-transfected or ARHGAP18-silencing HCCLM3 cells. (**C**) Transwell migration assay was carried out in CDKN2BAS-overexpressing cells after ARHGAP18 knockdown. (**D**) The expression of ARHGAP18 in cancer tissues and paired adjacent non-cancer tissues in the non-metastatic group was determined; (**E**) The expression of ARHGAP18 in cancer tissues and paired adjacent non-cancer tissues in the metastatic group was determined; (**F**) The correlation between CDKN2BAS transcript and ARHGAP18 mRNA was analyzed in 44 metastatic HCC tissues.

We then determined if the miR-153-5p/ARHGAP18 signaling contributed to CDKN2BAS-induced HCC cell migration. Transfection of miR-153-5p mimics significantly reduced the migration capacity of HCCLM3 cells (Figure 5A). Similar result was found following ARHGAP18 knockdown (Figure 5B). Moreover, knockdown of ARHGAP18 counteracted the promotional effect of CDKN2BAS on cell migration (Figure 5C). Additionally, in the metastatic group, ARHGAP18 expression was significantly up-regulated in cancer tissues compared with non-cancer tissues, while there was no significant difference in the non-metastatic group (Figure 5D-E). Furthermore, there was a significantly positive correlation between the expression of CDKN2BAS and ARHGAP18 (Figure 5F, R^2^ = 0.3417). Together, these results indicate that CDKN2BAS promotes cell migration via the miR-153-5p/ARHGAP18 signaling.

### CDKN2BAS inhibits EPI-induced apoptosis via activating MEK/ERK signaling

**Figure 6:**
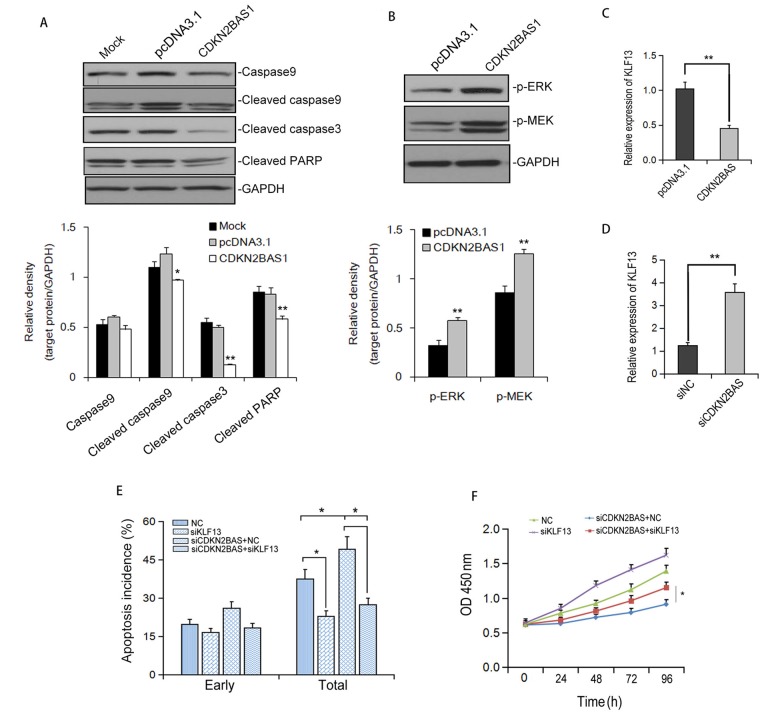
**CDKN2BAS down-regulates KLF13 expression and inhibits EPI-induced apoptosis.** (**A**) The expression of caspases and cleaved PARP after EPI treatment was detected by Western blot; (**B**) The effect of CDKN2BAS overexpression on the phosphorylation of ERK and MEK in HCCLM3 cells was detected by Western blot; (**C**-**D**) The effect of CDKN2BAS overexpression or knockdown on KLF13 mRNA expression was detected by qRT-PCR; (**E**) EPI–induced apoptosis was detected by flow cytometry in HCCLM3 cells transfected with CDKN2BAS siRNA and/or KLF13 siRNA; (**F**) Cell proliferation of HCCLM3 cells was detected by MTT after siRNA transfection.

To understand the mechanism of CDKN2BAS-mediated apoptosis inhibition, we first determined the effect of CDKN2BAS on the expression of apoptosis-related proteins. Western blot analysis showed that overexpression of CDKN2BAS significantly eliminated the protein levels of cleaved-caspase 9, cleaved-Caspase 3 and cleaved-PARP induced by EPI in HCCLM3 cells (Figure 6A). Additionally, overexpression of CDKN2BAS activated MEK/ERK signaling pathway (Figure 6B). Also, transfection of CDKN2BAS reduced the expression of KLF13, a potential negative regulator of MEK/ERK signaling pathway. In contrast, knockdown of CDKN2BAS up-regulated KLF13 protein level (Figure 6C-D). Knockdown of KLF13 markedly inhibited EPI-induced apoptosis and obviously eliminated the increased apoptosis induced by CDKN2BAS knockdown (Figure 6E). Moreover, silencing of KLF13 restrained the inhibitory effect of CDKN2BAS knockdown on the viability of HCC cells (Figure 6F). These results indicated that CDKN2BAS suppresses EPI-induced apoptosis via down-regulation of KLF13 and activation of MEK/ERK pathway.

## DISCUSSION

Increasing evidence supports that lncRNAs plays a major role in human physiological and pathophysiological processes [10,24]. LncRNA CDKN2BAS is a member of lncRNA family and functions in human pediatric breast cancer [21], brain tumor [22], and medulloblastoma predisposition [23]. In this study, using the microarray with metastatic and non-metastatic HCC tissues we found that the expression of lncRNA CDKN2BAS was significantly up-regulated in metastatic tumors. The expression of CDKN2BAS in most of the cancer tissues was also up-regulated compared with the adjacent non-cancer tissues. The upregulation of CDKN2BAS in HCC tissues indicated that CDKN2BAS may be essential in the progression and metastasis of HCC. Indeed, results from migration assay, scratch assay, MTT assay and colony formation assay *in vitro*, as well as the tumor xenograft model in nude mice *in vivo* demonstrated a critical role of CDKN2BAS in HCC metastasis and tumorigensis.

We further explored the potential mechanism of CDKN2BAS in promoting HCC metastasis. It has been shown that lncRNAs can act as a ceRNA by competitively binding with microRNA. Our data showed that CDKN2BAS has a role of ceRNA via acting as a miR-153-5p sponge, and it can improve the expression of miR-153-5p target gene ARHGAP18. miR-153-5p has been reported to play important roles in cancer metastasis, recurrence, and progression. ARHGAP18 is a member of RhoGAP gene family, which promotes GTP hydrolysis and inactivates RhoGTPase [25], but the effects of ARHGAP18 in cancer remain controversial. Aleskandarany *et al.* found that the aggregation of ARHGAP18 in the cell nuclei improved the prognosis of patients with breast cancer [26]. Another study of triple-negative breast cancer by Humphries *et al.* showed that ARHGAP18 could function as the oncogene by blocking RhoA signaling and reversing the inhibition of miR-200b on cell migration [27]. Our results demonstrated that ARHGAP18 plays an oncogenic role promoting tumorigenesis and metastasis in HCC. Moreover, we revealed that miR-153-5p/miR-153-5p signaling plays a causal role in CDKN2BAS-enhanced HCC metastasis.

## CONCLUSION

In conclusion, we have found that lncRNA CDKN2BAS plays an important role in the progression and metastasis of HCC. Importantly, CDKN2BAS/miR-153-5p/ARHGAP18 signaling axis may provide new clues for understanding the molecular mechanism of HCC progress and help to develop new diagnosis and treatment strategies.

## MATERIALS AND METHODS

### Clinical specimens

Cancer tissues and the paired adjacent non-cancer tissues in 85 patients who received primary HCC surgery during January 2012 to July 2014 were collected from Surgical Center, The First People’s Hospital of Wenling. All patients signed the informed consent. The study was approved by the ethical review committee of the First People’s Hospital of Wenling. In all patients, 44 patients had intrahepatic metastasis (tumors in the main branch of the portal vein); 41 patients had solitary tumors and without the occurrence of metastasis or recurrence during 2-year follow-up. The expression profiles of lncRNA and mRNA were obtained for 5 fresh frozen primary HCC tissues and their corresponding non-cancer tissues. All samples were immediately placed in liquid nitrogen and stored at -80°C.

### LncRNA microarray analysis

LncRNA Human Gene Expression Microarray V4.0 (CapitalBio Corp, Beijing, China) was utilized in this study. The double strand cDNA was synthesized, purified, and eluted. The complementary RNA was synthesized using T7 Enzyme Mix and the eluted dsDNA products. After amplification, cDNA was purified and labeled. CapitalBio BioMixerTM II Hybridization Station was used to cross the chip overnight. And then the chip was washed and scanned. Microarray images were transformed into point intensity value. The signal was directly output to the GeneSpring software after deducting the background value and carried out the quantile standardization. The data was further analyzed. Differential expressed lncRNA was chosen based on the following criteria: fold change > 2 and P < 0.05. The hierarchical clustering analysis was conducted on the differential expressed lncRNA.

### Cell culture and transfection

HCCLM3, SK-Hep-1, HUH7, MHCC97H, and L02 cells were cultured in DMEM medium containing 10% fetal bovine serum (FBS) and incubated in a humidified atmosphere of 5% CO_2_ at 37°C. pcDNA3.1-CDKN2BAS plasmid, control pcDNA3.1 plasmid, and siRNA (GenePharma, Shanghai, China) were transfected into cells using Lipofectamine 2000 reagent (Invitrogen, California, USA).

### Cell motility analysis

Cell motility was determined by the Transwell migration assay and the scratch assay. Briefly, 1 × 10^4^ cells were added into the upper chamber with polycarbonate microporous membrane (Millipore, Bedford, Massachusetts). After 24 h, cells transferred to the bottom of the membrane were fixed and stained with crystal violet. Scratch assay was carried out on the cell layer scratched by the pipette tip after transfection for 24 h. Images of the scratches were captured at 0 h and 48 h.

### MTT assay

Cells were seeded at a density of 4×10^3^/well in 96-well plates 24 h after transfection. At different time points, 10 μl MTT (5 mg/mL, Sigma, USA) was added to each well and cells were cultured for another 4 h. The samples were dissolved in DMSO after staining and the absorbance was measured at 450 nm.

### Colony formation assay

Cells were seeded at a density of 500/well in 6-well plates and cultured for 10 days to form cell colony. Then cells were washed twice with PBS and fixed with 4% polyoxymethylene for 15 min. Fixed cell colonies were stained with crystal violet for 10 min, photographed, and counted.

### Tumor xenograft models in nude mice

To detect the effect of CDKN2BAS on the growth and migration of HCC cells in vivo, the CDKN2BAS lentivirus vector was stably transfected to luciferase-labeled HCC cells. Inoculation of tumor cells into the nude mice were performed as previously described [19]. For tumor metastasis analysis, HCC cells were injected into the nude mice via the tail vein. Bioluminescence was observed by IVIS@ Lumina II system every week. For tumor growth evaluation, 1×10^7^ cells were injected subcutaneously into the right rear flank of mice. Tumor volumes were measured used with a caliper. After 4 weeks, the tumors in the mice were collected and tumor weight was measured. Animal experiments were approved by Experimental Animal Care and Use Committee of the First People’s Hospital of Wenling (Approval number: FPHW-14X03).

### Apoptosis analysis

Apoptosis was analyzed by flow cytometry with Annexin V-PI apoptosis detection kit. Cells after transfection were treated with EPI for 24 h. Cells were then collected and stained with Annexin V-PI (BD Biosciences PharMingen) according to the instructions. The early and late apoptotic cells were detected by flow cytometry after double staining.

### Luciferase reporter gene assay

The 3'-UTR of ARHGAP18 containing miR-153-5p responsive element was cloned into pGL4.13 luciferase reporter vector (ARHGAP18 wt-3’UTR). Luciferase mutant vector was obtained by mutating miR-153-5p binding site (ARHGAP18 mut-3’UTR). To confirm the relationship among CDKN2BAS, miR-153-5p, and ARHGAP18, ARHGAP18 wt/mut-3’UTR, together with miR-153-5p mimics/NC mimics, or pcDNA3.1-CDKN2BAS, pcDNA3.1-CDKN2BAS-mut/blank vectors were transfected into MHCC97H cells. Dual-Luciferase Reporter Assay System (Promega) was used to analyze the luciferase activity after transfection for 48 h.

### Statistical analysis

All data were presented as mean ± S.D. Student’s t-test was used to analyze the parameterized variables. Non-parametric variables were analyzed by chi-square test and Fisher’s exact test (2-tail). All the tests were repeated at least three times. *P* < 0.05 was considered as statistically significant.
